# The Effects of Nisin Treatment on the Phenylpropanoid and Physiological Mechanisms of Fresh-Cut Pumpkin

**DOI:** 10.3390/foods14050733

**Published:** 2025-02-21

**Authors:** Yuge Guan, Yan Sun, Ning Yuan, Rentao Zhang, Sainan Lu, Qianqian Li, Xinghua Lu, Linjiang Pang, Wenzhong Hu

**Affiliations:** 1 School of Food and Health, Zhejiang Agricultural and Forestry University, Hangzhou 311300, Chinahuaqianshua@163.com (X.L.);; 2 College of Life Science, Dalian Minzu University, Dalian 116600, China

**Keywords:** fresh-cut pumpkin, nisin, preservation quality, amino acid metabolism, phenylpropanoid metabolism

## Abstract

Pumpkin is rich in nutritional value, and it can be eaten as a vegetable or as a staple food, making it popular among modern consumers. However, after fresh cutting, pumpkins are susceptible to moisture loss, softening, microbial contamination, and browning, all of which significantly compromise their quality during storage. Therefore, it is essential to develop effective preservation techniques for maintaining the quality of fresh-cut pumpkins. Nisin, a safe natural preservative, has not yet been studied for use on fresh-cut pumpkins. This study examines the effects of nisin treatment on the quality of fresh-cut pumpkins and then explores preservation mechanisms based on physiological and metabolomic analysis. Results show that 0.4 g/L nisin treatment effectively delays surface browning without impacting odor and maintains microbial safety throughout storage. Additionally, nisin significantly enhances the activities of phenylalanine ammonia-lyase, cinnamate-4-hydroxylase, 4-coumarate-CoA ligase, and cinnamyl alcohol dehydrogenase, thereby promoting the accumulation of total phenols and carotenoids. The result of the Kyoto Encyclopedia of Genes and Genomes (KEGG) enrichment of differential metabolites between control and nisin-treated groups reveals that the most significant pathways affected by nisin treatment are amino acid metabolism and phenylpropanoid metabolism, which suggests that nisin enhances preservation by modulating phenylpropanoid metabolism and alleviating amino acid metabolism. This study provides a theoretical basis and offers new insights into improving the storage quality of fresh-cut pumpkins.

## 1. Introduction

Pumpkin (Cucurbita moschata (Duch. ex Lam.) Duch. ex Poiret) is a traditional food widely cultivated worldwide, belonging to the Cucurbitaceae family and classified as an annual climbing herb [1,2]. China is its largest producer, accounting for about one-third of the world’s pumpkin yield [3]. Due to being rich in nutrients and characterized by a soft, sweet taste, both consumed as a vegetable and staple, pumpkins are gaining popularity among consumers. However, their large size complicates peeling and seeding, while their time-consuming preparation limits their appeal to consumers. In recent years, the fresh-cut fruit and vegetable industry has experienced rapid growth, with ready-to-eat products becoming increasingly favored by consumers [4]. Consequently, processing pumpkins into convenient fresh-cut products through grading, washing, peeling, cutting, preservation, and packaging has gained popularity [5]. Fresh-cut pumpkin is not only 100% edible but can also effectively save the time of ordinary consumers before meal preparation, which is in line with the fast-paced lifestyle of contemporary people, greatly reduces the time cost of cooking, and meets consumers’ pursuit of fast food, light food, and safe food concepts [6]. Additionally, the centralized production of fresh-cut products minimizes kitchen waste, reducing urban waste and associated disposal costs, thus complying with environmental and food safety standards and presenting a broad market prospect [7]. However, fresh-cut pumpkins are susceptible to quality deterioration, including color-fading, microbial contamination, softening, and nutrient loss, resulting in accelerated decay [8,9]. Therefore, maintaining their quality and extending their shelf life are essential to the fresh-cut pumpkin industry.

Currently, preservation techniques for fresh-cut pumpkins can be classified into three main categories [10]. The first is physical preservation technology including low temperatures, heat treatment, ozone, high static pressure, modified-atmosphere packaging, etc., which primarily reduces weight loss, delays surface browning, and preserves nutritional quality by inhibiting browning-related enzyme activity and enhancing antioxidant capacity [11]. The second is chemical preservation technology, mainly including methyl jasmonate (MeJA), chitosan coating, ethylene, calcium, 1-methylcyclopropene (1-MCP), salicylic acid (SA), hydrogen sulfide (H_2_S), etc., which can improve the activity of antioxidant-related enzymes in fresh-cut pumpkin, reduce the content of reactive oxygen species (ROS), and slow down the occurrence of membrane lipid peroxidation, thereby maintaining quality [12]. The third is biological preservation technology, which refers to the use of natural extracts and antagonistic microorganisms to inhibit physiological activities and extend shelf life. This approach is highly efficient, cost-effective, safe, and environmentally friendly, garnering significant scientific attention [13]. For instance, Bacillus subtilis effectively suppresses respiration and weight loss while maintaining color by regulating pectinase, galacturonase, peroxidase (POD), and polyphenol oxidase (PPO) activities; at the same time, the growth of yellow berry fungus and pepper phytophthora on the surface is also inhibited, ensuring the microbial safety of fresh-cut pumpkin during storage [8].

Nisin is a bacteriocin derived from Lactococcus lactis subsp. Lactis, which has been used as a food preservative in the food industry since 1950. Nisin is listed as generally recognized as safe (GRAS) and was allowed to be used as a food preservative by the U.S. Food and Drug Administration (FDA) in 1980 [14,15]. In order to verify whether nisin has a preservation effect on fruits and vegetables, many scholars have conducted a series of studies. For example, Chen et al. studied the effect of using nisin in combination with citric acid on fresh-cut onions, finding that the levels of caffeic acid, vanillic acid, and ferulic acid increased, color was well preserved, and microbial counts remained below the detection threshold [16]. The combination of nisin with lactic acid, chitosan, and ε-polylysine significantly inhibited the respiration rate, ascorbic acid degradation, and microbial growth (including yeasts, molds, total colony counts, and coliforms) in fresh-cut carrots, while enhancing the total phenol content and phenylalanine ammonia-lyase (PAL) activity [17]. Meanwhile, it suppressed carrot whitening and significantly reduced lignin synthesis in fresh-cut carrots by inhibiting cinnamate-4-hydroxylase (C4H) and 4-coumarate-CoA ligase (4CL) activity [17]. Fernández et al. discovered that the combined treatment of nisin with green tea extract in fresh-cut beet leaves greatly increased the total phenol content and antioxidant capacity, maintained microbial safety, and consequently extended shelf life [18]. Małaczewska and Kaczorek-Łukowska reported that nisin treatment combined with ultrasound-assisted heat significantly enhanced the total phenolic content and antioxidant capacity of grape juice, preserved anthocyanin and flavonoid levels, effectively ensured microbial safety, and improved the storage quality of the juice [19]. Furthermore, nisin has been studied for its preservation effects on white mushrooms [20], fresh-cut lettuce [21], fresh-cut mango [22], and cucumber juice beverages [23]. However, there is no research yet on the impact of nisin on the quality of fresh-cut pumpkin; in particular, the regulatory mechanism of nisin on the quality of fresh-cut pumpkin is still unclear.

In this study, we used “Green Chestnut” pumpkin as the study subject to clarify the effect of nisin treatment on the quality, flavor, and microbial load of fresh-cut pumpkin over a 10-day storage period. Meanwhile, this study explored the changes in the total phenol and lignin contents, as well as the relationship between critical enzymes related to the phenylpropanoid metabolic pathway and intermediate metabolites. Moreover, metabolomics analysis was employed to identify significant differential metabolites following nisin treatment and to utilize bioinformatics for elucidating key metabolic pathways. Through the above results, this study aims to reveal the regulatory mechanisms of nisin on the quality of fresh-cut pumpkin and offers new insights for effective preservation methods.

## 2. Materials and Methods

### 2.1. Plant Materials and Pre-Treatment

Fresh “Green Chestnut” pumpkins were purchased from a local supermarket; those with a uniform shape, similar maturity, and an absence of pests or mechanical damage were selected and stored at 4 °C. The batches of pumpkins were washed and soaked in a 0.1% sodium hypochlorite (Tianjin Kemiou Chemical Reagent Co., Ltd., Tianjin, China) solution for 2 min [24]. After air-drying, the pumpkins were peeled, seeded, and cut into quarter-circle slices measuring 0.5 cm thick and 2 cm wide. The pumpkin slices were mixed evenly and then randomly divided into two groups, which were immersed in solutions of 0 or 0.4 g/L nisin (Shanghai Yuanye Chemical Reagent Co., Ltd., Tianjin, China) for 10 min. Moisture on the surface was gently wiped away with sterile gauze, and the samples were air-dried on a sterile workbench. The treated fresh-cut pumpkins were randomly placed in polyethylene trays (19.5 cm × 12.5 cm × 1.8 cm), wrapped in polyethylene film, and stored at 4 °C for 10 d, with samples collected every 2 d for analysis.

### 2.2. Appearance, Aroma, and Microbial Content Measurement

Photographs were taken every two days to record the appearance of the samples. The aroma of fresh-cut pumpkins was assessed using a portable electronic nose (Air Sense Portable Electronic Nose Pen3, Berlin, Germany). The procedure involved placing 2.5 g of a pumpkin sample in a 20 mL vial and allowing it to equilibrate at room temperature for 30 min before analysis. Instrument parameters included sensor cleaning for 60 s, sample preparation for 5 s, sensing the chamber flow rate of 200 mL/min, and detecting for 60 s. Data from the stable state between 56 and 58 s were selected for analysis, in conjunction with the electronic nose’s sensor performance (Table 1).

The total microbial count, as well as the number of molds and yeasts in the fresh-cut pumpkins, was determined using the plate counting method [25]. Samples were homogenized and diluted, and we then used Plate Count Agar (PCA), Violet Red Bile Dextrose Agar (VRBDA), and Potato Dextrose Agar (PDA) for measuring the total viable count, coliform count, and mold and yeast counts, respectively. The results of the total plate counts and mold and yeast contents were expressed as log (CFU/g).

### 2.3. Measurement of Lipoxygenase Activity and Malondialdehyde Content

Lipoxygenase (LOX) enzyme activity was measured in a 0.2 g frozen sample, following the LOX assay kit’s instructions (LOX-1-W, Suzhou Kemi Biotechnology Co., Ltd., Suzhou, China). Ultraviolet absorbance at 280 nm was measured for each sample (A1) and the control solution (A2), with the difference ΔA = A1 − A2 being calculated. One LOX enzyme unit was defined as an increase of 0.01 absorbance units per gram of tissue per minute at 25 °C.

The malondialdehyde (MDA) content was measured as per the kit’s instructions (MDA-1-Y, Suzhou Kemi Biotechnology Co., Ltd., Suzhou, China), based on the condensation of MDA with thiobarbituric acid (TBA) to produce a red compound with a maximum absorbance at 532 nm. The result of the MDA content was expressed as nmol/g.

### 2.4. Measurement of Total Phenols, Lignin, and Carotenoid Content

The total phenol content (TPC) was determined according to the Folin–Ciocalteu method described by a previous report with minor modifications [26]. We took 5 g samples mixed with 20 mL of 80% ethanol; after, it was homogenized in an ice bath and sonicated at 40 °C for 40 min. Then, the mixture was centrifuged for 20 min, and the supernatant was used for analysis. The supernatant (2 mL) was mixed with 2 mL Folin–Ciocalteu reagent, and after mixing thoroughly, 20 mL of 7% Na_2_CO_3_ solution was added, and deionized water was used to make up the volume to 50 mL. The mixture was left in the dark for 90 min before we measured the absorbance at 750 nm spectrophotometrically (Hitachi U-2800 Spectrophotometer, Tokyo, Japan). The TPC was expressed as mg/g, which was calculated with the gallic acid standard curve.

The lignin content was measured according to the procedure provided by the kit (MZS-1-G, Suzhou Kemi Biotechnology Co., Ltd., Suzhou, China) using a microplate reader (Hitachi U-2800 Spectrophotometer, Tokyo, Japan). Pumpkin samples were dried at 80 °C to constant weight and then ground and sieved through a 40-mesh sieve. About 2 mg of the samples was placed in a glass test tube. The difference between the absorbance of the sample (A1) and the blank control group (A2) at 280 nm was measured (ΔA = A1 − A2). The lignin content was expressed as mg/g and calculated based on the standard curve (y = 0.0694x + 0.0068, R^2^ = 0.9889).

The carotenoid content was measured following the method of Bahar Demircan et al. with slight modifications [27]. Briefly, we took 5 g of pumpkin sample, mixed with 20 mL of anhydrous ethanol, and extracted it in the dark with an ultrasonic wave for 8 min, centrifuged it at 4 °C and 12,000× *g* for 10 min, aspirated the supernatant, and repeated the extraction 4 times. The absorbance of the extract was measured at 450 nm and the carotenoid content was expressed as g/100 g based on the carotenoid standard curve.

### 2.5. Measurement of Phenylpropanoid Metabolism-Related Enzyme Activity

The determination of phenylalanine ammonia-lyase (PAL) activity was carried out according to the method reported by previous researchers [28]. Five grams of sample was mixed with 20 mL of extraction buffer (0.1 mol L^−1^, pH 8.8 borate-boric acid buffer) and centrifuged at 4 °C and 12,000× *g* for 30 min; then, the supernatant was collected for the determination of enzyme activity. The reaction system was composed of 0.5 mL of supernatant, 3 mL of 50 mmol L^−1^ pH 8.8 borate buffer, and 0.5 mL of 20 mmol L^−1^ L-phenylalanine. Absorbance at 290 nm was measured immediately (initial value OD_0_) and again after a 60 min incubation at 37 °C (final value OD_1_). An increase in the absorbance of 0.01 units per hour at 290 nm was defined as one PAL activity unit.

4-coumarate-CoA ligase (4CL) activity was measured based on modified methods from previous reports [29]. Five grams of pumpkin sample was mixed with 20 mL of pre-chilled extraction buffer (pH 8.0, 50 mmol L^−1^ Tris-HCl buffer containing 15 mmol L^−1^ β-mercaptoethanol, 0.15% PVP, 10% glycerol, 1 mmol L^−1^ PMSF, 10 μmol L^−1^ leupeptin, 5 mmol L^−1^ ascorbic acid, and 4 mmol L^−1^ MgCl_2_). The mixture was homogenized on ice and then centrifuged at 4 °C and 12,000× *g* for 30 min to collect the supernatant as the enzyme extract. For the activity assay, 0.5 mL of enzyme extract was combined with 0.05 mL of 0.6 mmol L^−1^ p-coumaric acid, 0.05 mL of 0.4 mmol L^−1^ CoA, 0.5 mL of 5 mmol L^−1^ ATP, and 2 mL of 5 mmol L^−1^ MgCl_2_. After 10 min of incubation, 100 μL of 6 mol L^−1^ HCl was added to terminate the reaction, and absorbance was measured at 333 nm. An increase of 0.01 absorbance units per minute was defined as one unit of 4CL activity (U).

The activities of cinnamyl-alcohol dehydrogenase (CAD), cinnamate-4-hydroxylase (C4H), and peroxidase (POD) were measured according to the manufacturer’s instructions using the respective kits (CAD-1-Y; C4H-1-Y; POD-1-Y; www.cominbio.com accessed on 18 May 2024). The determination of CAD activity was based on the principle that CAD catalyzes the conversion of cinnamyl alcohol and NADP^+^ to cinnamaldehyde and NADPH. The rate of NADPH production, measured at 340 nm, was used to indicate CAD activity. Similarly, C4H catalyzed the conversion of cinnamic acid and NADP^+^ to 4-coumarate and NADPH, with its activity determined at 340 nm based on the rate of NADPH production. The change in absorbance at 340 nm from the initial value (A_1_) to the final value after 5 min (A_2_) was calculated (ΔA = A_2_ – A_1_), and one activity unit was defined as the production of 1 nmol NADPH per gram of tissue per minute (U/g). The POD activity was assessed with a specific kit by monitoring the absorbance at 470 nm at two time points: 1 min (A_1_) and 2 min (A_2_). The difference in absorbance (ΔA = A_2_ − A_1_) was then calculated. One activity unit (U/g) was defined as an absorbance change of 0.005 per gram of tissue in a 1 mL reaction system per minute.

### 2.6. Metabolomics Analysis

#### 2.6.1. Metabolite Extraction

Metabolite extraction was performed with acidified methanol according to De Cheng et al. [30]. Briefly, 100 mg of powdered pumpkin sample was mixed with 0.12 mL of pre-chilled 50% methanol and then vortexed for 1 min. After extracting for 10 min at 25 °C, the mixture was centrifuged at 4000× *g* for 20 min and then the supernatant was filtered with a 0.22 μm organic membrane and transferred to a 96-well plate for LC-MS analysis. Additionally, an equal amount of each sample was used to prepare quality control (QC) samples by mixing 10 μL of the diluted solution.

#### 2.6.2. Analysis of Metabolites by Ultra-Performance Liquid Chromatography (UPLC)-Mass Spectrometry (MS)

The untargeted metabolomics analysis was performed using a UPLC-MS system (Thermo Fisher Scientific, Bremen, Germany) according to the procedures described by Li et al. (2018) [31] with some modifications. A reversed-phase ACQUITY UPLC T3 column (100 mm × 2.1 mm, 1.8 µm, Waters, Milford, CT, USA) was used for separation, with the column oven temperature set to 35 °C. The mobile phase comprised solvent A (water with 0.1% formic acid) and solvent B (acetonitrile with 0.1% formic acid) at a flow rate of 0.4 mL/min. A high-resolution tandem mass spectrometer (TripleTOF 5600 plus SCIEX, Cheshire, UK) detected the metabolites in both positive and negative ion modes with a maximum injection time of 100 ms, and fragmentation spectra were collected at a 17,500 resolution with a maximum injection time of 80 ms. QC samples were analyzed after every ten samples to assess the stability of the collection process. Raw data were converted to a readable format and then imported into XCMS software (XCMS online https://xcmsonline.scripps.edu/landing_page.php?pgcontent=mainPage, Scripps Research Institute, New York, NY, USA) for peak extraction, retention time correction, quality control, and aggregate ion analysis. Secondary mass spectrometry data were matched against the KEGG (KEGG online, http://www.kegg.jp/) database for metabolite identification and annotation.

### 2.7. Statistical Analysis

Data processing was performed using Microsoft Excel (2020), and graphs were plotted with Origin 8.0. Statistical significance was tested in IBM SPSS 25.0 (*p* < 0.05). Supervised PLS-DA was conducted through metaX to discriminate the different variables between groups. Differential ions were filtered by criteria: a ratio ≥ 2 or ≤1/2; a *p*-value < 0.05; the variable important for the projection value ≥ 1.

## 3. Results

### 3.1. Effects of Nisin Treatment on Edibility Quality and Microbial Safety of Fresh-Cut Pumpkin

#### 3.1.1. Effect of Nisin Treatment on Appearance

**Figure 1:**
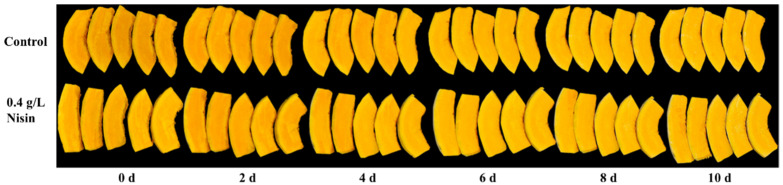
Effects of nisin treatment on appearance quality in fresh-cut pumpkins.

The appearance quality of fruits and vegetables directly affects consumers’ willingness to buy products, so it is an important evaluation indicator for evaluating the edible quality of fresh-cut pumpkins during storage. In our prior study, we observed that the whiteness index (WI) of fresh-cut pumpkins increased during storage [32]. However, treatment with 0.4 g/L nisin significantly curbed this rise throughout the storage period. As depicted in Figure 1, the color of fresh-cut pumpkins progressively turned whiter over time. In the control group, slight browning appeared by day 8 and became more pronounced by day 10. In contrast, the group treated with nisin showed only minimal browning by day 10. This indicates that nisin effectively preserved the visual quality of fresh-cut pumpkins.

#### 3.1.2. Impact of Nisin Treatment on Aroma

**Figure 2:**
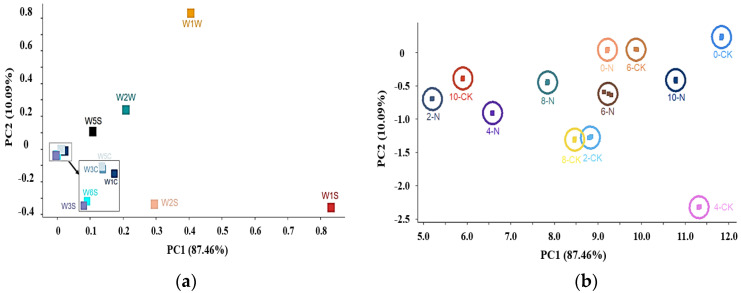

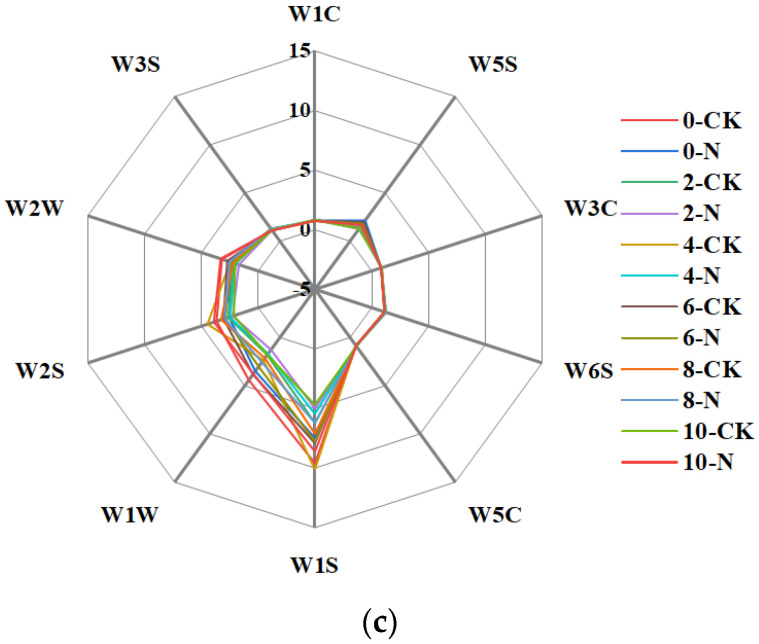
Effects of nisin treatment on loading analysis plot (**a**), principal component analysis plot (**b**), and odor characteristics (**c**) of fresh-cut pumpkins.

To further analyze the impact of nisin on the flavor of fresh-cut pumpkins, electronic nose technology was employed. Linear discriminant analysis (LDA) was used to assess the contribution of different sensors, identifying the main volatile components in fresh-cut pumpkins. As shown in Figure 2a, the first principal component (PC1) accounted for 87.46% of the variance, and the second principal component (PC2) accounted for 10.09%, totaling 97.55%. This indicates that these two components captured the primary characteristics of the fresh-cut pumpkins. Notably, sensor W1S contributed most to PC1, followed by sensors W1W, W2S, and W2W. The highest contributions to PC2 came from sensors W1W and W2W. Therefore, the primary aroma components in the fresh-cut pumpkins likely included short-chain alkanes like methane and inorganic sulfides, as well as alcohols, ethers, aldehydes, ketones, and aromatic compounds.

Additionally, principal component analysis (PCA) was used to reduce the dataset to two principal components (PC1 on the *X*-axis and PC2 on the *Y*-axis). As shown in Figure 2b, samples clustered closely in the plot indicate similar properties, and when the two components contributed over 95%, they effectively encompassed all original sample information. The contribution rates of PC1 and PC2 were 87.46% and 10.09%, respectively, confirming that these components captured the key characteristics of the fresh-cut pumpkins. Although the treatment groups do not overlap, they are closely spaced, indicating that the electronic nose effectively differentiated the fresh-cut pumpkin samples stored for different durations and nisin did not affect the odor components of the fresh-cut pumpkin.

As shown in Figure 2c, among the ten sensors, W1S, W1W, W2W, and W2S had aroma response values (G/G_0_) greater than 2.0, while the other six sensors were less responsive, with values below 2.0. This suggests that aroma changes in the fresh-cut pumpkins during storage were mainly influenced by compounds like short-chain alkanes, inorganic sulfides, alcohols, ethers, aldehydes, ketones, and aromatic substances, consistent with Figure 2a. Therefore, the radar chart further indicates no significant changes in aroma characteristics between the treatment and control groups, confirming that nisin treatment did not affect the fresh-cut pumpkins’ aroma components.

#### 3.1.3. Impact of Nisin Treatment on Microbial Content

**Figure 3:**
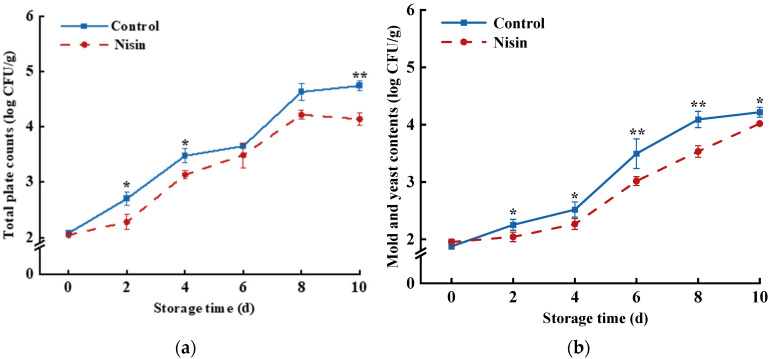
Effects of nisin treatment on number of bacterial colonies (**a**) and total amount of mold and yeast (**b**) in fresh-cut pumpkins, the * indicates a statistically significant difference (*p* < 0.05) and the ** indicates a statistically significant difference (*p* < 0.01).

Fresh-cut fruits and vegetables are highly susceptible to microbial contamination due to wounding stress during processing, which leads to quality deterioration [33]. As shown in Figure 3a,b, the total bacterial count, mold, and yeast levels gradually increased in both groups during storage; however, the nisin-treated group consistently exhibited lower colony counts than the control group. This indicates that nisin treatment effectively suppressed bacterial, mold, and yeast growth in the fresh-cut pumpkins, thereby enhancing microbial safety. Similar results have been reported in studies on fresh-cut jackfruit [34]. The action of nisin on microbial cell membranes may cause the leakage of small molecules, such as amino acids, K^+^, and ATP, disrupting membrane potential and pH gradient, ultimately leading to cell lysis and death, which further helps maintain microbial safety during storage and prolongs shelf life for fresh-cut produce [35].

### 3.2. Impact of Nisin Treatment on Lipoxygenase Activity and Malondialdehyde Content

**Figure 4:**
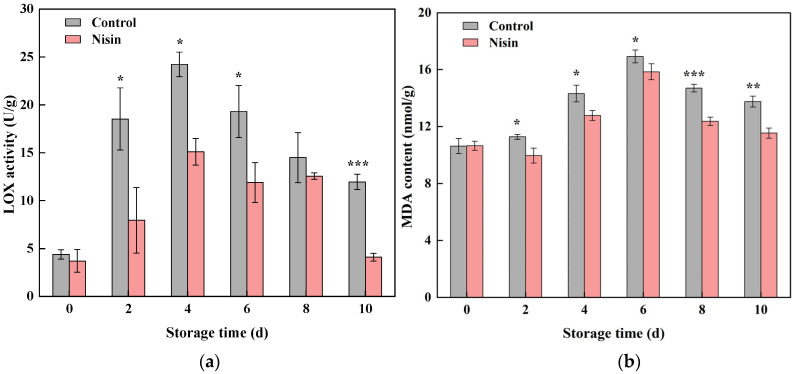
Effects of nisin treatment on lipoxygenase (LOX) activity (**a**) and malondialdehyde (MDA) content (**b**) in fresh-cut pumpkins, the * indicates a statistically significant difference (*p* < 0.05) and the ** indicates a statistically significant difference (*p* < 0.01), and the *** indicates a statistically significant difference (*p* < 0.001).

Mechanical damage to fruits and vegetables generates numerous reactive oxygen species (ROS), as peroxyl radicals extract electrons from the phospholipid bilayer of cell membranes, disrupting membrane integrity and initiating lipid peroxidation [36]. The lipoxygenase (LOX) enzyme is crucial in this process, catalyzing fatty acid conversion into harmful by-products, with malondialdehyde (MDA) being one of the most representative peroxidation products [37]. As shown in Figure 4a, LOX activity in the fresh-cut pumpkins increased during the first four days of storage and then gradually declined, with the nisin-treated pumpkins consistently displaying lower LOX activity than the controls. Similarly, the MDA content showed a trend of first increasing and then decreasing (Figure 4b), and the nisin treatment could significantly reduce the MDA content of the fresh-cut pumpkins, indicating that nisin treatment could inhibit the occurrence of membrane lipid peroxidation. Previous studies also showed that nisin treatment inhibited LOX activity in cucumber juice more effectively than low-pressure or pasteurization methods [23], highlighting its preservative role in slowing the aging of fresh-cut produce.

### 3.3. Impact of Nisin Treatment on Carotenoid Content

**Figure 5:**
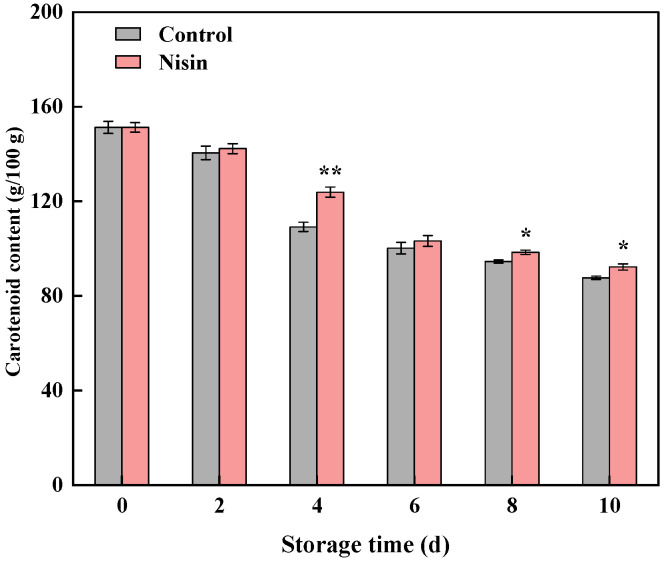
Effects of nisin treatment on carotenoid content in fresh-cut pumpkins, the * indicates a statistically significant difference (*p* < 0.05) and the ** indicates a statistically significant difference (*p* < 0.01).

Carotenoids, a primary source of vitamin A and the key antioxidant in pumpkins, protect against oxidative damage but are highly unstable during processing and storage [38]. They also serve as major pigments, but they can easily isomerize or bind with proteins, resulting in color loss and reduced sensory quality [39]. Figure 5 shows that the carotenoid content in the fresh-cut pumpkins declined during storage; however, nisin treatment slowed this decline from days 4 to 10, suggesting that it helped preserve nutritional content. This result aligns with browning observations [32], further supporting nisin’s role in preserving the color of fresh-cut pumpkins.

### 3.4. Effects of Nisin Treatment on Phenylpropanoid Metabolism in Fresh-Cut Pumpkin

#### 3.4.1. Impact of Nisin Treatment on Total Phenols and Lignin Content

**Figure 6:**
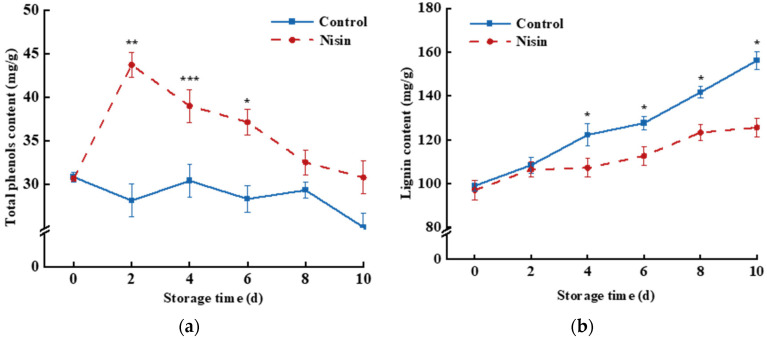
Effects of nisin treatment on total phenols (**a**) and lignin content (**b**) in fresh-cut pumpkins, the * indicates a statistically significant difference (*p* < 0.05), the ** indicates a statistically significant difference (*p* < 0.01), and the *** indicates a statistically significant difference (*p* < 0.001).

Phenolic substances are secondary metabolites of plants and substrates for lignin synthesis. When fruits and vegetables are in the cutting process, phenylpropanoid metabolism accumulates phenolic substances to resist wounding stress and reduce the damage of ROS [36]. As shown in Figure 6a, total phenol content in the control group fluctuated, while the nisin group had significantly higher levels, peaking at 1.55 times that of the control group on day 2, which suggests that nisin treatment could promote phenol content in the fresh-cut pumpkin.

Lignin, a product of phenylpropanoid metabolism, aids in resisting the infection of microorganisms but also contributes to the whitening of fresh-cut pumpkins [40]. Figure 6b shows that lignin content in fresh-cut pumpkins increased over time, and the lignin content in the nisin treatment group was significantly lower than that in the control group from the 4th to 10th days. After 4, 6, 8, and 10 days, the lignin content of the nisin-treated group was 12.29%, 11.81%, 12.77%, and 19.87% lower than that of the control group, respectively. This study found that the total phenol content increased first and then decreased during storage, while the lignin content increased during the whole storage. This may have been because the fresh-cut pumpkin was subjected to cutting stress, which activated the phenylpropanoid metabolic pathway, increased the total phenol content, and then converted it into lignin, resulting in a decrease in the total phenol content [41]. However, the increase in lignin content was beneficial to enhancing the resistance, but it also affected the degree of whitening of the cut surface of the fresh-cut pumpkin. In this study, we found that nisin treatment could effectively reduce the accumulation of lignin, thereby reducing the occurrence of whitening and maintaining the quality of the fresh-cut pumpkins.

#### 3.4.2. Impact of Nisin Treatment on Key Enzyme Activities in Phenylpropanoid Metabolism

**Figure 7:**
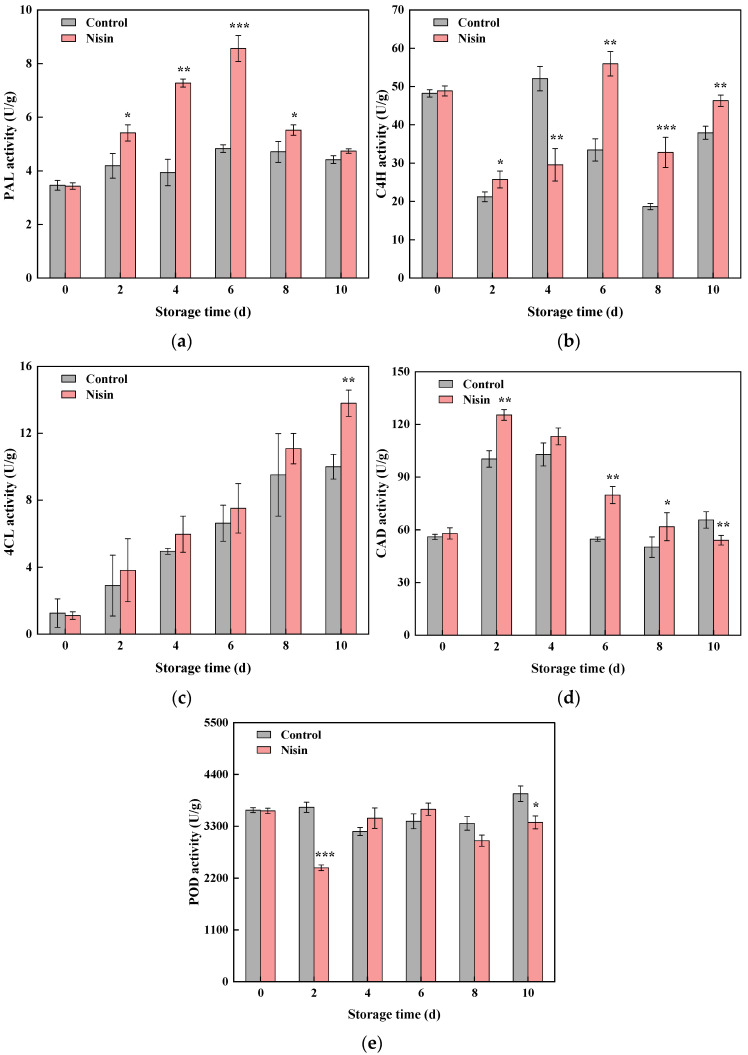
Effects of nisin treatment on phenylalanine ammonia-lyase (PAL) (**a**), cinnamate-4-hydroxylase (C4H) (**b**), 4-coumarate-CoA ligase (4CL) (**c**), cinnamyl-alcohol dehydrogenase (CAD) (**d**), and peroxidase (POD) (**e**) activity in fresh-cut pumpkins, the * indicates a statistically significant difference (*p* < 0.05), the ** indicates a statistically significant difference (*p* < 0.01), and the *** indicates a statistically significant difference (*p* < 0.001).

The phenylpropanoid pathway initiates with L-phenylalanine deamination to form cinnamic acid, regulated by the PAL enzyme. Subsequent conversions involve C4H synthesizing p-hydroxycinnamic acid and 4CL forming 4-coumarate-CoA, which contributes to the synthesis of more phenolic substances [42]. CAD acts as the rate-limiting enzyme in lignin monomer formations [43], while POD catalyzes the final step in lignin synthesis [44]. As shown in Figure 7a, PAL activity in the control group remained stable, whereas that in the nisin group peaked from days 0 to 6 and then gradually declined, remaining significantly higher than that in the control group. During storage on day 6, the PAL activity of the nisin group was 1.77 times greater than that of the control group. Figure 7b shows that both groups exhibited fluctuations in C4H activity, with that in the nisin group being consistently higher from days 6 to 10. During storage, 4CL activity in the fresh-cut pumpkins increased (Figure 7c), with the nisin group consistently displaying higher levels than the control group. The above results indicate that it was precisely because nisin upregulated the activity of C4H and 4CL that the total phenol content in the nisin group was always higher than that in the control group. Figure 7d shows that CAD activity in the nisin group remained higher during storage, peaking on day 2. The POD activity trends differed between groups: In the control group, POD activity increased from days 0 to 2 and again from days 4 to 10, with the values on days 2 and 10 surpassing those in the nisin group. In contrast, the nisin treatment sustained an upward trend in POD activity from days 2 to 6, peaking on day 6 before declining (Figure 7e). Overall, the nisin treatment effectively enhanced key enzyme activities in phenylpropanoid metabolism, accelerating phenolic compound synthesis and strengthening resistance in the fresh-cut pumpkins.

### 3.5. Impact of Nisin Treatment on Metabolites

To investigate how nisin regulates quality in fresh-cut pumpkins, this study used non-targeted metabolomics to detect unbiased dynamic changes in metabolic ions. We then found the identified differential metabolites to further clarify the nisin treatment’s effects on metabolic pathways, offering theoretical support and new insights for preserving fresh-cut pumpkin [45].

#### 3.5.1. Identification of Metabolites in Fresh-Cut Pumpkin Treated with Nisin

**Figure 8:**
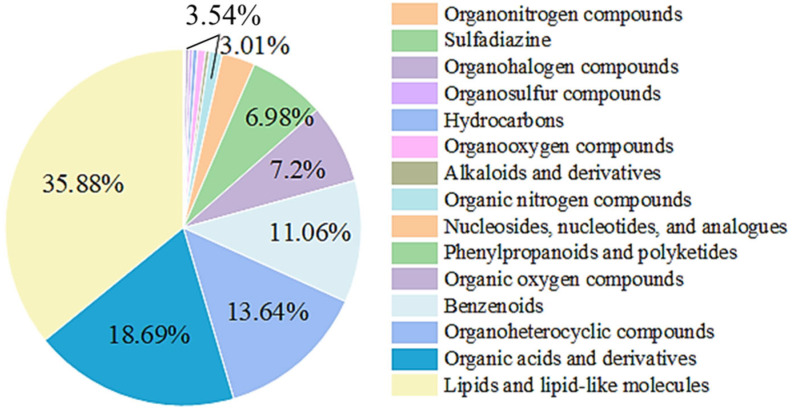
Superclass classification of secondary metabolites in fresh-cut pumpkins.

The identification of secondary metabolites is shown in Figure 8. Results indicate that the most abundant metabolites in the fresh-cut pumpkin were lipids and lipid-like molecules, totaling 334 and accounting for 35.88%. This was followed by organic acids and derivatives (174, 18.69%); organic heterocyclic compounds (127, 13.64%); phenolic compounds (103, 11.06%); organic oxides (67, 7.2%); phenylpropanoids and polyketides (65, 6.98%); nucleosides, nucleotides, and analogs (28, 3.01%); and organic nitrogen compounds (11, 1.18%). Other categories included minor proportions of alkaloids and derivatives, organic oxides, hydrocarbons, organic halogen and sulfur compounds, and sulfanilamide.

**Figure 9:**
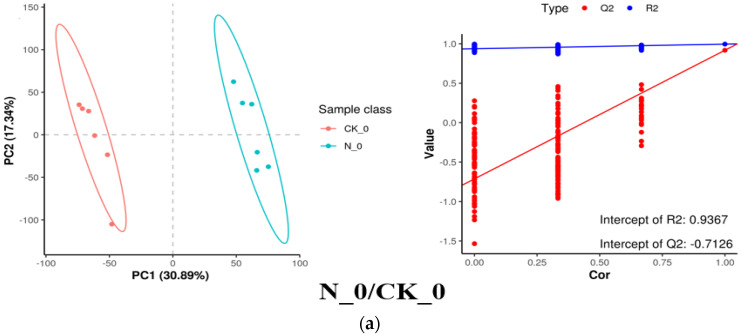

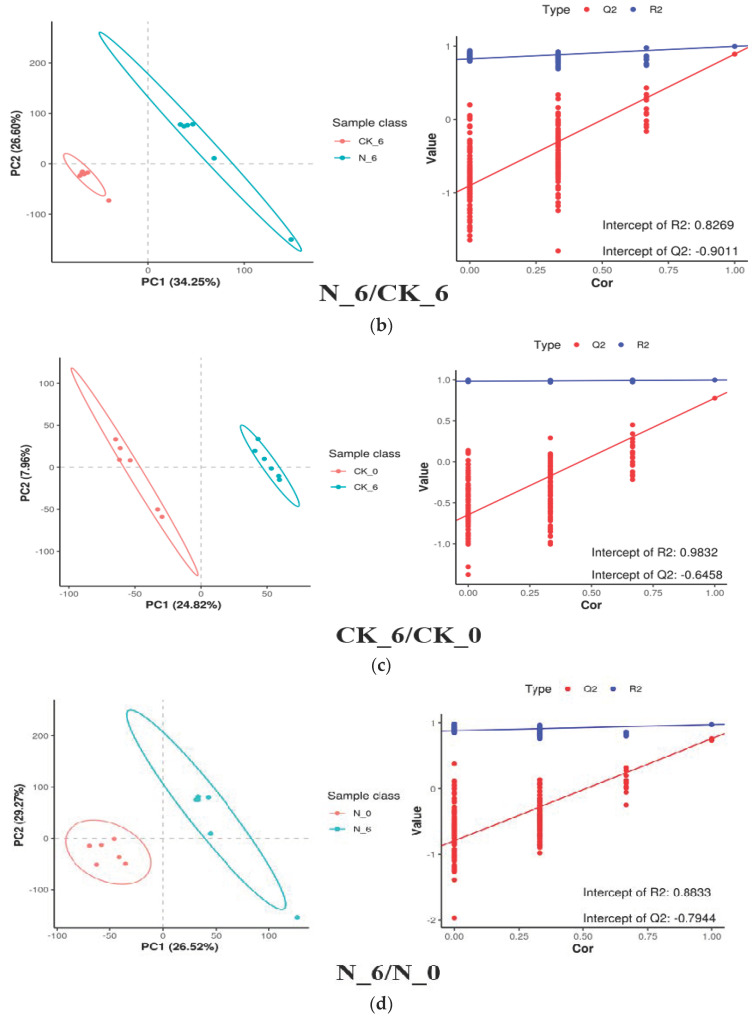
The metabolic ion partial least squares–discriminant analysis (PLS-DA) model score chart and replacement test chart of N_0/CK_0 (**a**), N_6/CK_6 (**b**), CK_6/CK_0 (**c**), and N_6/N_0 (**d**) in fresh-cut pumpkins.

To validate the separation of the nisin-treated samples, partial least squares–discriminant analysis (PLS-DA) analysis was conducted. As shown in Figure 9, the PLS-DA model score plots and permutation test plots for N_0/CK_0, N_6/CK_6, CK_6/CK_0, and N_6/N_0 groups reveal significant metabolic differences, with high predictive and explanatory power. Moreover, the PLS-DA model had good predictive and explanatory capabilities, and the permutation test results were all Q2 < 0, indicating that the model was not overfitting and the differential metabolite analysis was relatively accurate.

#### 3.5.2. Analysis of Differential Metabolites in Fresh-Cut Pumpkin Treated with Nisin

**Figure 10:**
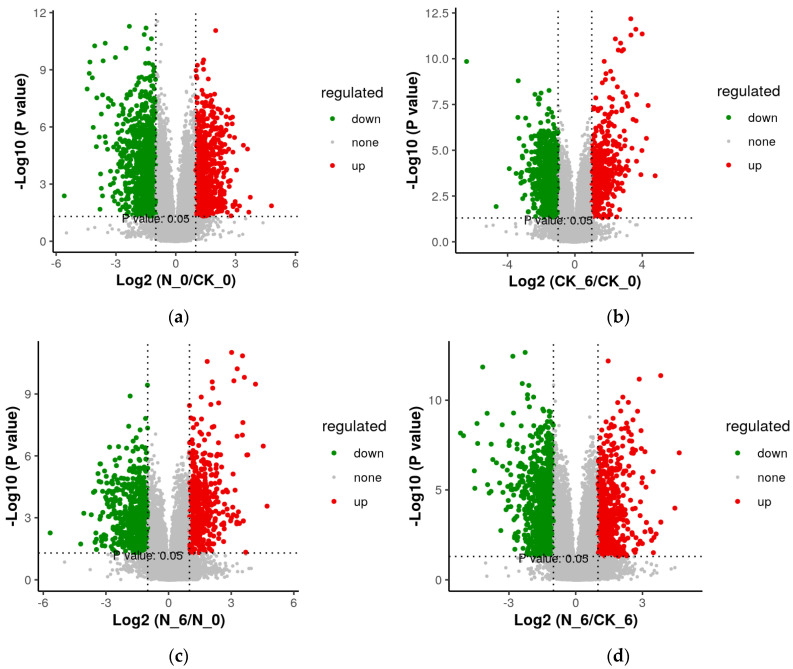
Volcanic maps of different metabolic ions between the N_6/CK_0 groups (**a**), CK_0/CK_0 groups (**b**), N_6/N_0 groups (**c**), and N_6/CK_6 groups (**d**) in fresh-cut pumpkins.

As shown in Table 2 and Figure 10, compared to the CK_0 group, the N_0 group had 804 up-regulated metabolites and 1430 down-regulated metabolites and the CK_6 group had 502 up-regulated metabolites and 1101 down-regulated metabolites. Compared to the N_0 group, the N_6 group had 550 up-regulated metabolites and 691 down-regulated metabolites. Compared to the CK_6 group, the N_6 group had 750 up-regulated metabolites and 1138 down-regulated metabolites. Through the volcano plot analysis, it can be seen that there were a large number of differential metabolites between the nisin group and the control group and between different storage periods.

**Figure 11:**
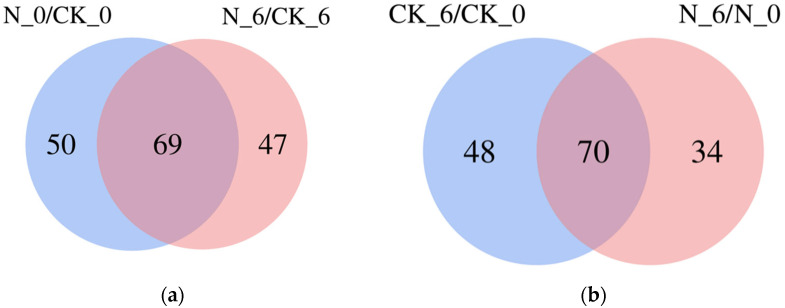
Venn diagrams of the N_0/CK_0 and N_6/CK_6 groups (**a**) and CK_6/CK_0 and N_6/CK_0 groups (**b**) in the fresh-cut pumpkins.

To further examine the types of differential metabolites, a Venn diagram (Figure 11a) is used to identify common differential metabolites between the N_0/CK_0 and N_6/CK_6 groups, identifying 69 shared metabolites, mainly comprising lipids and lipid-like molecules (23), phenylpropanoids and polyketides (14), organic acids and derivatives (11), organic heterocycles (6), organic oxides (5), and phenolic compounds (2). The Venn diagram (Figure 11b) shows that the CK_6/CK_0 and N_6/N_0 groups shared 70 common differential metabolites, mainly including organic acids and derivatives (28), lipids and lipid-like molecules (18), organic heterocycles (8), phenolic compounds (4), phenylpropanoids and polyketides (3), organic oxides (2), and alkaloids and derivatives (1). These shared differential metabolites were responsive to storage time and may be key metabolites involved in fresh-cut pumpkin senescence.

#### 3.5.3. Enrichment Analysis of Differential Metabolites in KEGG Pathways

**Figure 12:**
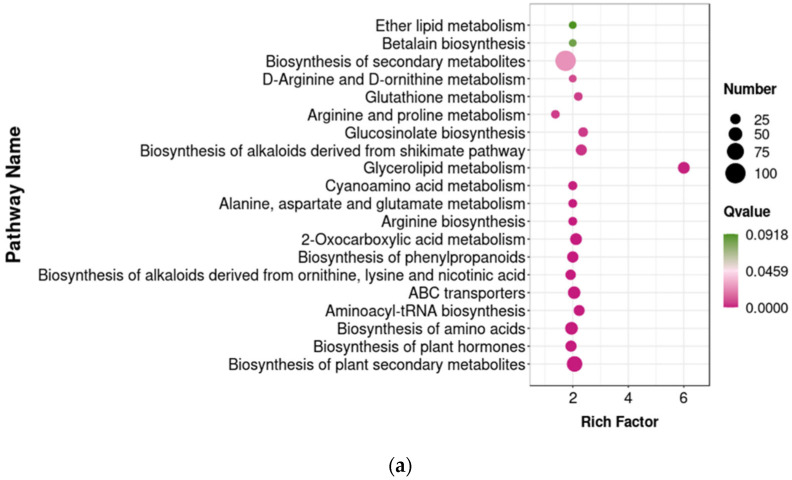

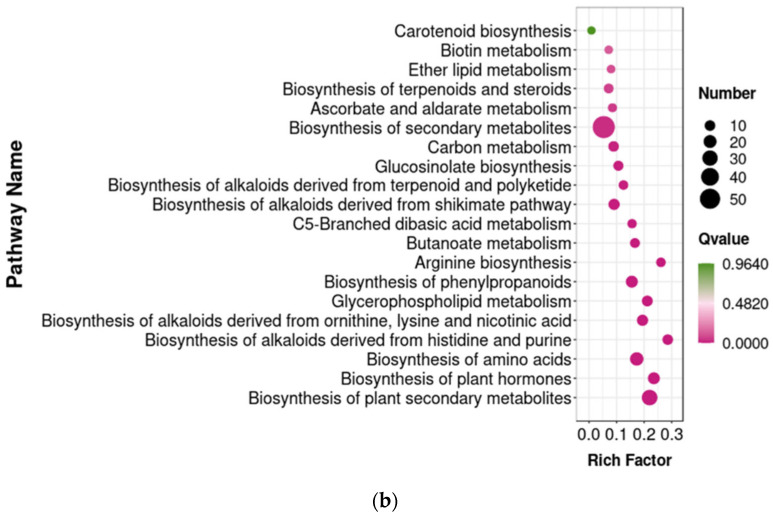
The common differential metabolite enrichment pathways between the N_0/CK_0 and N_6/CK_6 groups (**a**) and between the CK_6/CK_0 and N_6/N_0 groups (**b**) in fresh-cut pumpkins.

To explore the functional roles of various metabolites, KEGG pathway enrichment analysis was conducted on differential metabolites in fresh-cut pumpkin after 0 d and 6 d of storage. Figure 12a shows that common differential metabolites of N_0/CK_0 and N_6/CK_6 were enriched in 35 KEGG pathways. The top 20 significantly enriched metabolic pathways included the biosynthesis of plant secondary metabolites, plant hormones, amino acids, and aminoacyl-tRNA; ABC transporters; the biosynthesis of alkaloids (ornithine, lysine, niacin); phenylpropanoids; glycerophospholipid, phenylalanine, oxalic acid, and arginine metabolism; purine metabolism; alanine, aspartate, and glutamate metabolism; linoleic acid and cyanogenic amino acid metabolism; glycerolipid metabolism; aldehyde acids and dicarboxylates metabolism; and alkaloid biosynthesis via the shikimic acid pathway.

As the storage time increased, the fresh-cut pumpkin quality deteriorated, suggesting that common differential metabolite pathways in the CK_6/CK_0 and N_6/N_0 groups represented primary pathways in quality degradation. The common differential metabolites of the CK_6/CK_0 and N_6/N_0 groups were enriched in 20 KEGG pathways (Figure 12b). The common differential metabolite pathways, in order of significance, were the biosynthesis of plant secondary metabolites, plant hormones, amino acids, histidine, and purine alkaloids; alkaloids from ornithine, lysine, and niacin; phenylpropanoids; glycerophospholipid metabolism; arginine biosynthesis; butanoate metabolism; C5 branched dicarboxylic acids; alkaloids via the shikimic acid pathway; terpenes and polyketide alkaloids; glucosinolates; carbon metabolism; and ascorbic acid metabolism, among others.

#### 3.5.4. Analysis of Key Metabolic Pathways Involving Differential Metabolites

In summary, the common differential metabolites between the two comparison groups showed the most significant differences in the phenylalanine metabolism and phenylpropanoid biosynthesis pathways, with 20 and 12 differential metabolites identified in amino acid biosynthesis and phenylpropanoid metabolism, respectively. These findings indicate that phenylalanine and phenylpropanoid pathways played crucial roles in regulatory mechanisms in the fresh-cut pumpkins, warranting further analysis.

**Figure 13:**
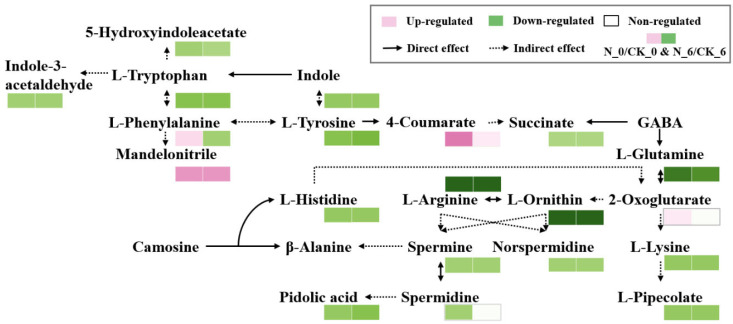
Pathways related to amino acid metabolism in fresh-cut pumpkins.

After cutting damage, secondary metabolic pathways in fruits and vegetables are activated, with amino acids serving as essential precursors for synthesizing secondary metabolites and playing a vital role in the plant [46]. As shown in Figure 13, after 0 d, the nisin-treated group had decreased levels of succinic acid, lysine, arginine, glutamine, ornithine, tryptophan, tyrosine, histidine, spermidine, piperine, indole, indole-3-aldehyde, spermine, hydroxyproline, and 5-hydroxy indole acetic acid, as well as decreased levels of keto-pentanoate, phenylalanine, phenyl ethyl nitrile, and p-coumaric acid, compared to the control group. This suggests that nisin treatment had a significant regulatory effect on the amino acid metabolism of the fresh-cut pumpkin. By day 6, the overall amino acid levels in the nisin-treated group were lower than those in the control group, with phenylalanine levels in the nisin group also lower than in the control group. Phenylalanine is a key metabolite linking primary metabolism (e.g., the tricarboxylic acid cycle and glycolysis) to secondary metabolism, such as phenylpropanoid pathways, and serves as a precursor for phenolic compound synthesis. This may be due to nisin treatment accelerating phenolic compound synthesis in fresh-cut pumpkin, converting phenylalanine to secondary metabolites, and leading to lower phenylalanine levels in the nisin group. This suggests that nisin treatment influences fresh-cut pumpkin storage quality by regulating multiple metabolic pathways.

**Figure 14:**
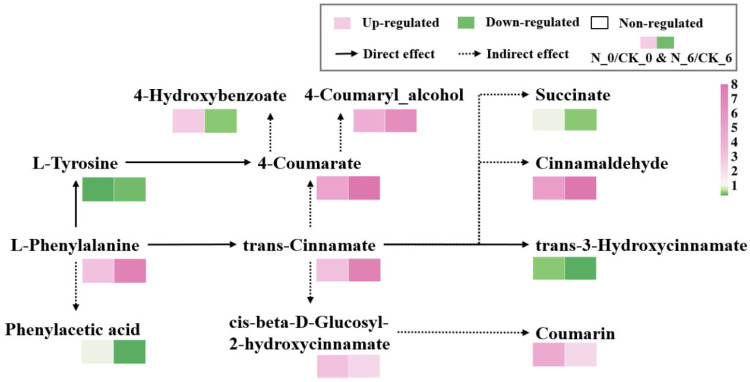
Metabolic pathways of phenylpropane in fresh-cut pumpkins.

Phenylpropanoid metabolism is the main pathway for synthesizing secondary metabolites, such as phenolic compounds, which have antioxidant properties, enhance stress resistance, and serve as lignin precursors [47]. Nisin treatment positively influenced phenylpropanoid metabolism in the fresh-cut pumpkin. As shown in Figure 14, after 0 d, the nisin group exhibited significantly elevated levels of phenylalanine, trans-cinnamic acid, p-coumaric acid, cinnamaldehyde, p-coumarin, coumarin, 4-hydroxybenzoate, and cis-β-D-glucosyl-2-hydroxycinnamate compared to the control group. After six days, differences in most other metabolites, except for p-coumaric acid and 4-hydroxybenzoate, increased continually. In N_6/CK_6, the relative contents of succinate and phenylacetate decreased, which may be because nisin treatment downregulated the phenylalanine content on the 6th day of treatment. Phenylalanine can not only be converted into tyrosine and trans-cinnamic acid during metabolism but also indirectly produces phenylacetate [48]. The above results indicate that nisin delayed whitening by regulating the phenylpropanoid metabolic pathway and inhibiting the amino acid metabolic pathway, thereby reducing the damage of wounding stress, maintaining the storage quality, and improving the preservation effect of the fresh-cut pumpkin during storage.

## 4. Conclusions

The treatment with 0.4 g/L nisin could effectively maintain the appearance quality of fresh-cut pumpkins. On the 8th day of storage, the total colony count in the nisin-treated group was 0.61 log CFU/g lower than that in the control group, and the amount of mold and yeast was 12.5% lower than that in the control group, indicating that nisin effectively maintained the edible quality and microbial safety of the fresh-cut pumpkin. Meanwhile, the nisin treatment reduced LOX enzyme activity, leading to a 16.15% decrease in MDA levels compared to the control group on day 8, effectively delaying membrane lipid peroxidation. In addition, the nisin treatment increased the activity of key enzymes in the phenylpropanoid metabolic pathway, such as PAL, C4H, 4CL, and CAD, accelerated the synthesis of phenolic substances, and increased the total phenol content of the nisin-treated group to 1.55 times that of the control group. It also reduced the lignin content by regulating POD activity (12.29–19.87% lower than that in the control group), which not only maintained the total phenol content (1.55 times higher than control) of the fresh-cut pumpkins but also delayed the lignification process of the fresh-cut pumpkins.

Based on non-targeted metabolomics technology, we found that nisin delayed whitening by regulating the phenylpropanoid metabolic pathway and inhibiting the amino acid metabolic pathway, thereby maintaining the storage quality and improving the preservation effect of the fresh-cut pumpkin during storage. Therefore, 0.4 g/L nisin treatment is an effective preservation method that could maintain the edible quality and microbial safety of fresh-cut pumpkins.

## Figures and Tables

**Table 1:** The different sensors of the electronic nose, Pen3, corresponding to the type of aroma.

Sensor Number	Sensor Name	Performance Description
S1	W1C	Aromatic compounds
S2	W5S	Nitrogen oxides
S3	W3C	Ammonia and aromatic compounds
S4	W6S	Hydrogen
S5	W5C	Aliphatic aromatic compounds
S6	W1S	Short-chain alkanes like methane
S7	W1W	Inorganic sulfides
S8	W2S	Alcohols, ethers, aldehydes, and ketones
S9	W2W	Aromatic compounds and organic sulfides
S10	W3S	Long-chain alkanes

**Table 2:** Effect of nisin treatment on differential metabolites in fresh-cut pumpkins.

Comparison Group	Positive Ions			Negative Ions			Total
	Total	Up	Down	Total	Up	Down	
N_0/CK_0	1324	506	818	910	298	612	2234
N_6/CK_6	1263	527	736	625	223	402	1888
CK_6/CK_0	1088	380	708	515	122	393	1603
N_6/N_0	909	406	503	332	144	188	1241

## Data Availability

The original contributions presented in the study are included in the article, further inquiries can be directed to the corresponding authors.
